# MetaLAFFA: a flexible, end-to-end, distributed computing-compatible metagenomic functional annotation pipeline

**DOI:** 10.1186/s12859-020-03815-9

**Published:** 2020-10-21

**Authors:** Alexander Eng, Adrian J. Verster, Elhanan Borenstein

**Affiliations:** 1 Department of Genome Sciences, University of Washington, Seattle, WA 98195 USA; 2 Present Address: Bureau of Food Surveillance and Science Integration, Food Directorate, Health Canada, Ottawa, ON K1A 0K9 Canada; 3 Blavatnik School of Computer Science, Tel Aviv University, 6997801 Tel Aviv, Israel; 4 Sackler Faculty of Medicine, Tel Aviv University, 6997801 Tel Aviv, Israel; 5 Santa Fe Institute, Santa Fe, NM 87501 USA

**Keywords:** Metagenomics, Functional annotation, Pipeline, Distributed computing

## Abstract

**Background:**

Microbial communities have become an important subject of research across multiple disciplines in recent years. These communities are often examined via shotgun metagenomic sequencing, a technology which can offer unique insights into the genomic content of a microbial community. Functional annotation of shotgun metagenomic data has become an increasingly popular method for identifying the aggregate functional capacities encoded by the community’s constituent microbes. Currently available metagenomic functional annotation pipelines, however, suffer from several shortcomings, including limited pipeline customization options, lack of standard raw sequence data pre-processing, and insufficient capabilities for integration with distributed computing systems.

**Results:**

Here we introduce MetaLAFFA, a functional annotation pipeline designed to take unfiltered shotgun metagenomic data as input and generate functional profiles. MetaLAFFA is implemented as a Snakemake pipeline, which enables convenient integration with distributed computing clusters, allowing users to take full advantage of available computing resources. Default pipeline settings allow new users to run MetaLAFFA according to common practices while a Python module-based configuration system provides advanced users with a flexible interface for pipeline customization. MetaLAFFA also generates summary statistics for each step in the pipeline so that users can better understand pre-processing and annotation quality.

**Conclusions:**

MetaLAFFA is a new end-to-end metagenomic functional annotation pipeline with distributed computing compatibility and flexible customization options. MetaLAFFA source code is available at https://github.com/borenstein-lab/MetaLAFFA and can be installed via Conda as described in the accompanying documentation.

## Background

The analysis of the functional capacities of microbial communities has become an important component of microbiome-based studies, providing novel insights into associations between the gut microbiome and host conditions such as depression [22], autism [18], and type 2 diabetes [16]. Such functional profiles are generally obtained via shotgun metagenomic sequencing and subsequent functional annotation. This functional annotation can be performed either by assembling reads into contigs and mapping detected open reading frames to annotated gene sequences, or by directly mapping individual reads to annotated gene sequences [17]. Assembly-based approaches can provide certain benefits by enabling the reconstruction of metagenomic assembled genomes (MAGs), but this assembly process can be incredibly challenging and prohibitively time and memory intensive. Additionally, these efforts often result in highly fragmented assemblies due to factors such as uneven sequencing depth across different genomes and high strain-level sequence diversity [7]. By comparison, assembly-free, or read-based, annotation approaches can offer a more practical and accessible option due to lower resource requirements and an avoidance of the assembly problem. The pipeline we present here utilizes this latter, read-based annotation approach.

To facilitate such read-based annotation processes, various pipelines, including HUMAnN2 [6], MG-RAST [10], eggNOG-mapper [8], SUPER-FOCUS [19], and YAMP [23], have been recently introduced. The standard workflow of these pipelines involves taking FASTQ or FASTA files as inputs, mapping reads to a database of microbial gene sequences, annotating reads with the functional capacities that have previously been associated with those genes, and eventually producing functional profiles at one or more levels of descriptive resolution. These pipelines, however, often lack one or more critical features essential for modern, high-throughput, complete, distributed, and computationally-intensive functional annotation, such as the ability to process unfiltered sequencing data, native integration with distributed computing systems, and/or adequate options for workflow customization (Table 1).

**Table 1:** Comparison of read-based metagenomic annotation pipeline features

Feature	MG-RAST	SUPER-FOCUS	eggNOG-mapper	HUMAnN2	YAMP	MetaLAFFA
Metagenomic functional annotation	✓	✓	✓	✓	✓	✓
Uses DIAMOND for read alignment	✓	✓	✓	✓	✓	✓
Read pre-processing	✓				✓	✓
Ortholog aggregation to broader functional categorizations	✓	✓	✓	✓	✓	✓
Available as a web service	✓		✓			
Native integration with distributed computing systems					✓	✓
Automatic continuation from intermediate steps after interruption				✓	✓	✓
Convenient incorporation of new pipeline steps					✓	✓
Universal single-copy gene-based abundance normalization via MUSiCC						✓

Here, we describe MetaLAFFA, a new functional annotation pipeline that addresses these shortcomings. MetaLAFFA avoids requiring users to separately run common pre-processing steps by incorporating various quality control measures into the pipeline. MetaLAFFA is also designed to easily and effectively integrate with compute cluster management systems, allowing users to take full advantage of available computational resources and distributed, parallel data processing. Finally, MetaLAFFA offers a convenient interface for configuring pipeline operation, providing users with extensive customization options that include the choice of which pipeline steps to perform and the operating parameters for individual steps.

## Implementation

### Default MetaLAFFA workflow

**Fig. 1:**
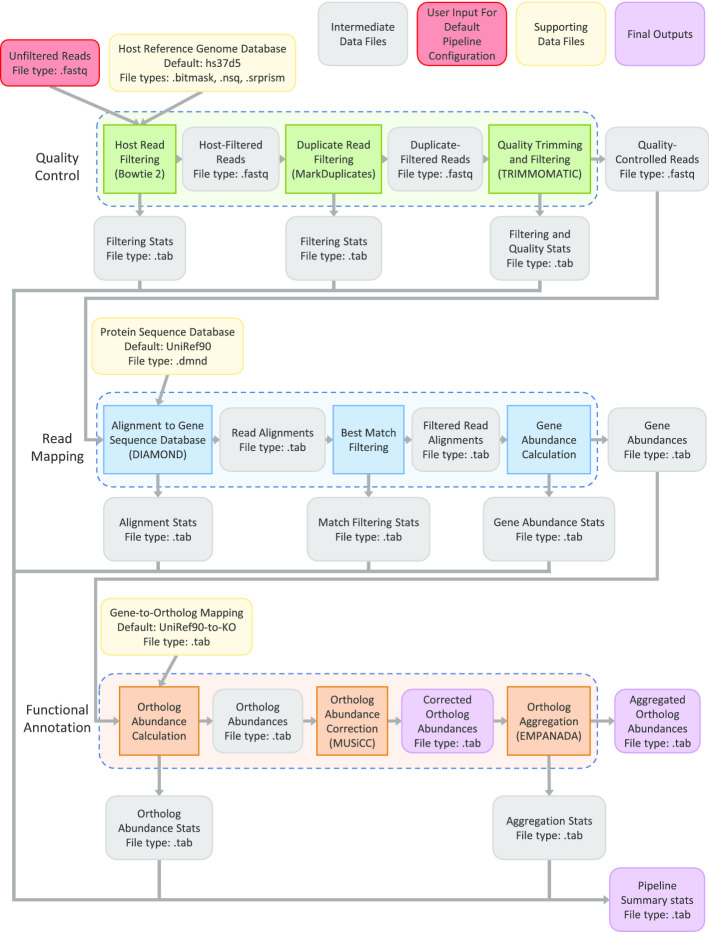
Flowchart of the default MetaLAFFA workflow. The default MetaLAFFA workflow consists of three phases, quality control (top), read mapping (middle), and functional annotation (bottom). This flowchart outlines the individual processing steps taken in each phase (colored rectangular boxes), the intermediate outputs of these steps (grey rounded boxes), supporting data files required for specific steps (yellow rounded boxes), user-provided input to MetaLAFFA (red rounded box), and the final outputs of the pipeline (purple rounded boxes). Third-party tools used in the default pipeline workflow are indicated in parentheses for their associated processing steps. File types of all inputs, outputs, and supporting data files are indicated by file suffix

In its default configuration, MetaLAFFA performs metagenomic annotation in three basic phases: quality control, read mapping, and functional annotation (Fig. 1). Broadly, the quality control phase aims to remove unwanted or low-quality reads from the input shotgun metagenomic data via common pre-processing operations. Next, the read mapping phase aligns the quality-controlled reads to a sequence database of microbial genes and calculates the abundance of each gene. Finally, the functional annotation phase translates these gene abundances into classifications of community functional capacities and their abundances within the community’s metagenome. In this section, we will elaborate on the exact steps performed by MetaLAFFA in its default configuration, including the default choices for tools and databases, and possible alterations users can make to fit their specific use cases.

MetaLAFFA’s quality control phase (Fig. 1, highlighted in green) was inspired by the Human Microbiome Project (HMP) [20] protocol, though specific methodology has been updated to reflect current best practices. While it utilizes some component tools similar to KneadData, MetaLAFFA does not employ KneadData to better compartmentalize each step and better enable user configuration and customization of individual components. First, MetaLAFFA removes host reads by mapping reads to the hs37d5 human genome reference with decoy sequences available from the 1000 genomes project [1] using Bowtie 2 [12] and then discarding any reads identified as human. Depending on the project, users can substitute alternative “host” databases to remove contaminants from different host organisms. Next, MetaLAFFA removes artificial duplicates by identifying duplicate reads using MarkDuplicates from the PICARD toolset [5] and then discarding those reads. The final step in the quality control phase is quality trimming and filtering, which removes low-quality bases from the ends of reads and then discards reads that are too short. This is performed using Trimmomatic [2] and the MAXINFO trimming criterion. After this quality control phase, users should be left with high-quality microbial reads that can serve as the basis for profiling community metagenomic content.

The read mapping phase (Fig. 1, highlighted in blue) begins with aligning reads to the UniRef90 database of protein sequences using DIAMOND [3], a core tool for rapid sequence alignment in most metagenomic annotation procedures. Other databases can be substituted for the UniRef90 database depending on the goals of the annotation project. For example, using a database like CARD, which contains a collection of antibiotic resistance genes [15], can allow users to focus specifically on profiling abundances of these antibiotic resistance genes within their community of interest. Similar to HUMAnN2 [6] and other pipelines, paired-end reads are mapped separately during the read mapping phase to avoid overly penalizing pairs where only one half of the pair aligns to a gene. After aligning reads, MetaLAFFA scans these matches to identify the best match (or best matches if there are ties) for each read in the database. This *best match* strategy was shown to yield high specificity in functional profiling [4], though users can choose alternative strategies to increase sensitivity at the cost of specificity. For example, MetaLAFFA also provides the option for a *best N matches* strategy, where a read is associated with the top N genes that the read mapped to. This strategy may be desirable if, for instance, the user is concerned that long reads may span adjacent genes. After refining matches from the alignment step, gene abundances are calculated by summing up the number of reads that mapped to each gene. If a read matches equally well to multiple genes, then that read contributes a fractional count distributed evenly across the abundances of each of those genes. This results in a gene abundance profile for each sample, which can be translated into more interpretable classifications of community functional capacities in MetaLAFFA’s third phase.

Finally, MetaLAFFA performs its functional annotation phase (Fig. 1, highlighted in orange). The first step in this phase is to calculate the abundances of genes with similar functional capacities, or functional orthologs. By default, these associations between genes and functional orthology groups come from the annotations in the UniRef90 database, mapping genes to KEGG orthology groups, or KOs [9]. If users choose a different database to map reads against (e.g. CARD), then users will need to update the gene-to-ortholog mapping appropriately. Similar to calculating gene abundances in the previous phase, MetaLAFFA does this by summing up the abundance of genes that belong to each group of functional orthologs. If a gene is associated with multiple orthology groups, its abundance is distributed evenly between the abundances of each of those orthology groups. The resulting functional profile is then corrected using MUSiCC [13] to convert read counts of orthologs into the average copy number per genome of each functional ortholog. Alternatively, users can set MetaLAFFA to leave functional profiles in terms of relative abundances. Finally, MetaLAFFA aggregates ortholog abundances into abundances of broader functional classifications using EMPANADA [14]. This tool aggregates KO abundances into KEGG pathway (and module) abundances based on the supporting evidence for the presence of each pathway. Users may need to change the ortholog-to-broader-classification mapping, or skip it all together, if they wish to use a different database for mapping reads to genes. The output of the functional annotation phase, and the final output from MetaLAFFA are the KEGG KO-, module-, and pathway-level profiles of community functional capacities.

After each step in the default MetaLAFFA pipeline is completed, MetaLAFFA also generates a table of summary statistics for that step. For example, during the steps in the quality control phase, these tables summarize how many reads were discarded from each sample, the new average base quality of each sample, and the average read length of each sample post-quality trimming. For later steps, these summary statistics include how many reads were successfully aligned to the gene database, how many matches remained when reduced to best matches, and how many unique functional orthologs were present in each sample. Once MetaLAFFA has completed its functional annotation, these summary statistics are combined into a single table summarizing the pipeline’s operation.

### Workflow management and distributed computing integration via Snakemake

MetaLAFFA is implemented in Snakemake [11], a Python-based workflow management framework that is specifically designed for bioinformatics analysis pipelines. Snakemake determines dependencies between different steps of a pipeline based on the expected inputs and outputs of each step, and ensures that later steps are only run once their inputs become available. One benefit of this approach is that Snakemake automatically detects when the expected outputs of a step are missing, halts the pipeline, and allows the user to simply resume operation from the most recent successfully completed steps after they address any issues that led to the failure.

Another useful feature of Snakemake is its ability to compartmentalize pipeline operations. Snakemake can split steps in the pipeline into independent jobs where, for example, each job processes a separate sample. MetaLAFFA takes advantage of this option to separate the quality control and read mapping of multiple FASTQ files into sample-specific jobs, rather than trying to process them all as a single operation. This compartmentalization means that if MetaLAFFA fails when processing a single sample (e.g. host filtering fails because the input FASTQ was malformed), any samples that were successfully processed prior to the failure will be recognized as successfully processed. Consequently, MetaLAFFA will avoid unnecessarily re-processing those samples once the cause of the failure has been addressed and the user resumes MetaLAFFA operation.

Finally, Snakemake has built-in capabilities to take advantage of parallel processing and distributed computing, which allows MetaLAFFA users to make the best use of their available computational resources. Specifically, Snakemake automatically determines which steps (and individual jobs when steps are split into separate jobs) are independent and can be run in parallel. Then Snakemake will use available cores if being run locally, or an available distributed computing environment (e.g. HTCondor, SGE, SLURM) to run independent jobs in parallel. This allows MetaLAFFA users to take full advantage of any available compute clusters and can enable the expedited annotation of large shotgun metagenomic datasets.

### MetaLAFFA configuration and customization

MetaLAFFA provides convenient pipeline configuration and customization options for both novice and advanced users. There are two main components that define pipeline configuration: a text file defining the overall pipeline workflow (i.e. how each step feeds into one or more subsequent steps) and a Python module that organizes general pipeline options and step-specific settings into separate submodules.

The workflow file enables users to make several modifications to the MetaLAFFA workflow and should be amenable to those with little or no programming experience. For example, setting a later step to use user-provided data as input will cause MetaLAFFA to skip prior steps during operation. This may be helpful if the user has already used quality control tools to process their data and would prefer to skip to MetaLAFFA’s read mapping phase. The workflow file is also used to designate special qualities to steps in the pipeline, including: which steps take user-provided files as initial input, which steps produce important final output files, which steps generate summary statistic tables, and which steps produce intermediate files that can be safely deleted after they’ve been used. Users can also modify this configuration file to reorder steps in the pipeline if so desired (e.g. perform host read removal after quality trimming and filtering) by changing how steps feed into each other.

The Python configuration module controls the rest of MetaLAFFA operation and can be used by both naïve and savvy users. Specifically, users with zero programming experience can still access this module to modify basic configuration options. The step-specific submodule that controls the read mapping step (“map_reads_to_genes.py”) offers a good example of this basic customization. For instance, this submodule sets the DIAMOND [3] operating parameters that will be used when mapping reads to genes, including the alignment method, the top percentage of best matches to keep, the E-value cutoff for saved matches, and the alignment sensitivity. Each of these parameters can be modified within the submodule by opening the file in a text editor and changing the appropriate value (e.g. finding where the “evalue_cutoff” variable is defined and changing its value from 0.001 to 1). Operating parameters for all steps in the pipeline can be modified in a similar manner.

Importantly, the values of parameters that can change a step’s output (e.g. different settings for DIAMOND’s sensitivity parameter) are tracked via output folder and file names. This helps users keep track of the specific MetaLAFFA configuration that produced a particular set of functional profiles. Additionally, this system leverages Snakemake’s usage of workflow specification through input and output naming patterns to enable users to more quickly experiment with different operating parameters. Specifically, if MetaLAFFA is run once under a specific configuration and the user then wants to rerun MetaLAFFA after changing parameters in various pipeline steps, MetaLAFFA will begin running from the earliest step for which parameters have been changed, rather than from the beginning of the entire pipeline. This is supported thanks to Snakemake’s ability to identify, based on output folder and file names, which intermediate outputs need to be newly generated.

Via the Python configuration module, Python-savvy users can further customize the actual operations run during each step in the pipeline. Returning to the read mapping step as an example, users can alter the behavior of this pipeline step by modifying the default function for step operations. This function tells MetaLAFFA how to run DIAMOND on indicated input files and passes DIAMOND the specified operating parameters mentioned above. Users can add code to this function that will be run in addition to the basic read alignment performed with DIAMOND, but they can also make more involved modifications such as changing which aligner MetaLAFFA uses for read mapping. Furthermore, users can add new steps to the pipeline, with existing step submodules serving as templates.

## Results

For a practical example of MetaLAFFA operation, we used MetaLAFFA in its default configuration to functionally annotate 4 stool samples (SRS011061, SRS011134, SRS011239, and SRS012273) from the HMP [20]. These samples ranged in size from 90 million reads to 130 million reads. Initial formatting of the input data and operation of MetaLAFFA to annotate these samples required very little effort, including:

1. Expanding the downloaded compressed sample directories.
2. Compressing individual read files to save disk space.
3. Modifying the suffixes of the forward, reverse, and singleton read filenames to match default MetaLAFFA expectations (“R1.fastq”, “R2.fastq”, and “S.fastq” respectively).
4. Creating a new MetaLAFFA project directory using the associated script.
5. Moving read files into the new project’s “data/” directory.
6. Running “./MetaLAFFA.py” from the command line

Since these data files are post-HMP quality control, minimal reads were discarded from each sample during MetaLAFFA’s quality control phase. The percentage of reads that had a match in the UniRef90 [21] database varied from 53% to 78% across samples, with unique gene matches ranging from 1.5 million to 2.7 million across samples. The resulting functional profiles contained 3.9–5.6 thousand unique KOs, 304–367 unique modules, and 135–141 unique pathways in each sample. The resulting KO-, module-, and pathway-level profiles, as well as a full summary of operating statistics for this MetaLAFFA run can be found in Additional file 1: Tables S1–S4.

## Conclusions

MetaLAFFA is an end-to-end functional annotation pipeline that incorporates several important features for efficient, high-throughput functional annotation of shotgun metagenomic data. It makes use of standard tools for shotgun metagenome processing and functional annotation to allow out-of-the-box operation for a wide audience, while also providing a convenient customization interface that allows users to tailor the pipeline to their specific needs. Implemented using Snakemake, MetaLAFFA can take advantage of extensive parallelization, making use of either local or distributed computing resources. Taken together, this combined convenience, customizability, and high-throughput nature of MetaLAFFA should increase the accessibility of shotgun metagenome functional annotation, enabling a larger audience to participate in exploring the functions of diverse microbial communities.

## Availability and requirements

*Project name* MetaLAFFA.

*Project home page* http://borensteinlab.com/software_metalaffa.html.

*Operating systems* Mac and Linux.

*Programming language* Python and Snakemake.

*Other requirements* Python 3.6 or greater, Conda 4.8 or greater, and Snakemake 3.13.3 or greater.

*License* GNU General Public License v3.0.

*Any restrictions to use by non-academics*: None.

## Supplementary information

**Additional file 1: Tables S1, S2, S3 and S4**. Example output tables generated by annotating 4 HMP samples using MetaLAFFA.

## Data Availability

The MetaLAFFA homepage can be found at http://borensteinlab.com/software_metalaffa.html. The code and documentation are both available on GitHub at https://github.com/borenstein-lab/MetaLAFFA. MetaLAFFA is available for installation via Conda. Example HMP data can be downloaded from https://hmpdacc.org/hmp/HMASM/.

## Abbreviation

HMP: Human microbiome project
